# CRF-based models of protein surfaces improve protein-protein interaction site predictions

**DOI:** 10.1186/1471-2105-15-277

**Published:** 2014-08-13

**Authors:** Zhijie Dong, Keyu Wang, Truong Khanh Linh Dang, Mehmet Gültas, Marlon Welter, Torsten Wierschin, Mario Stanke, Stephan Waack

**Affiliations:** Institute of Computer Science, University of Göttingen, Goldschmidtstr. 7, 37077 Göttingen, Germany; Institute of Bioinformatics, University of Göttingen, Goldschmidtstr. 1, 37077 Göttingen, Germany; Institut für Mathematik und Informatik, Walther-Rathenau-Str. 47, 17487 Greifswald, Germany

## Abstract

**Background:**

The identification of protein-protein interaction sites is a computationally challenging task and important for understanding the biology of protein complexes. There is a rich literature in this field. A broad class of approaches assign to each candidate residue a real-valued score that measures how likely it is that the residue belongs to the interface. The prediction is obtained by thresholding this score.

Some probabilistic models classify the residues on the basis of the posterior probabilities. In this paper, we introduce pairwise conditional random fields (pCRFs) in which edges are not restricted to the backbone as in the case of linear-chain CRFs utilized by Li *et al.* (2007). In fact, any 3D-neighborhood relation can be modeled. On grounds of a generalized Viterbi inference algorithm and a piecewise training process for pCRFs, we demonstrate how to utilize pCRFs to enhance a given residue-wise score-based protein-protein interface predictor on the surface of the protein under study. The features of the pCRF are solely based on the interface predictions scores of the predictor the performance of which shall be improved.

**Results:**

We performed three sets of experiments with synthetic scores assigned to the surface residues of proteins taken from the data set *PlaneDimers* compiled by Zellner *et al.* (2011), from the list published by Keskin *et al.* (2004) and from the very recent data set due to Cukuroglu *et al.* (2014). That way we demonstrated that our pCRF-based enhancer is effective given the interface residue score distribution and the non-interface residue score are unimodal.

Moreover, the pCRF-based enhancer is also successfully applicable, if the distributions are only unimodal over a certain sub-domain. The improvement is then restricted to that domain. Thus we were able to improve the prediction of the *PresCont* server devised by Zellner *et al.* (2011) on *PlaneDimers*.

**Conclusions:**

Our results strongly suggest that pCRFs form a methodological framework to improve residue-wise score-based protein-protein interface predictors given the scores are appropriately distributed. A prototypical implementation of our method is accessible at http://ppicrf.informatik.uni-goettingen.de/index.html.

## Background

Protein-protein interactions are constitutive of almost every biological process. The ability to identify the residues that form the interaction sites of these complexes is necessary to understand them. In particular, it is the basis for new therapeutic approaches to treat diseases [1, 2].

A great deal of work has been done on developing in-silico prediction methods. As already observed by Zhou *et al.* [3], these methods can be subdivided with respect to the kind of mathematical foundation invoked and with respect to the features or characteristics of the protein used.

### Residue-wise score-based prediction methods

Let *x*_*r*_ be the data relevant for a residue *r* in a given protein chain. These methods then employ a function *f*(*x*_*r*_,**λ**), where **λ** are some coefficients which have been learned through the training. The value of *f*(*x*_*r*_,**λ**) then determines, whether *r* is rated as an interface or not. The linear regression method [4, 5], the scoring function method [6–11], the neural network method [12–17], and the support vector machine method [18–25] are of this kind.

### Probabilistic methods

Let **X** be the data relevant for a protein chain, where these data are assumed to stem from a random source thus obeying a random distribution. **X**, which alternatively is called the observation, typically includes the structure. The label sequence of the residues **Y** that classifies each individual residue either as interface or as non-interface is assumed to be random, too. Typically, probabilistic methods use the conditional probability distribution  (**Y** | **X**) to determine a classification y* of the residues of maximal posterior probability  (y* | **x**). Naive Bayesian methods [26], Bayesian network methods [27], hidden Markov models (HMMs) [26], and linear-chain Conditional Random Fields taking the backbone as underlying graphical structure [28] fall in this category. Using posterior decoding on the basis of the forward-backward algorithm, both HMMs and CRFs are residue-wise score-based prediction methods, where the binary decision is made by thresholding the posterior probabilities of classifying the residues as interface.

### Notations

We use Latin uppercase letters when referring to random aspects of the objects denoted by them. In contrast, lowercase letters denote arbitrarily chosen but fixed objects. In this context boldface letters indicate vectors, the corresponding non-boldface letters their coefficients.

The vast majority of methods use the 3D structure of the target protein chain in form of a PDB file as input [4–13, 15, 17–21, 23–25]. However, a few methods are not requiring a 3D structure and rather use sequences only [14, 16, 22]. We here consider the problem with a given 3D structure of the target protein chain. Sequence-based input may include a multiple sequence alignment of related proteins from which, for example, sequence conservation can be inferred. When the 3D structure of an unbound binding partner is also available, protein-protein docking methods can be applied. This has also been exploited to provide feedback from docking to the more specific problem of interface prediction [29]. We here consider the case where the binding partner’s 3D structure is not given. Nor requires the presented method the sequence of the binding partner. Albeit, we tested on homodimers only as we here rather focus on our new method rather than on features or types of proteins. The protein features used for interface prediction in the literature are reviewed in the Methods section as far as we make use of them in this article.

Most of the current studies for predicting interaction sites of proteins that use a probabilistic method are restricted by treating the residues of the proteins as independent vertices. Li *et al.* have taken the backbone neighborhood into account thus modeling the protein as a sequence [28] using what can be called a *line CRF* or linear-chain CRF. The features they define on the label pair of two backbone neighbors have the effect of smoothing the predicted labels along the protein sequence. Decisive is, however, that they were the first who used conditional random fields (CRFs) for interface prediction. CRFs in turn have come into use for solving sequence labeling problems due to Lafferty *et al.* [30]. See [31] for an overview. From the mathematical point of view they take advantage of the fact that they model the conditional probability  (**Y** | **X**) rather than the joint probability  (**Y**, **X**). Recently there has been an explosion of interest in conditional random fields (CRFs) with successful applications. It has been shown that CRFs have the abilities for solving sequence labeling problems like part-of-speech tagging (POST) [32] and natural language processing [33]. Furthermore in the web extraction problem, in which the web-sites are modeled as two dimensional grid graphs, CRFs perform well [34]. One of their outstanding benefits over many other statistical models is that a CRF can easily describe the dependencies of observations.

As proteins are folded into three dimensional structures, spatial relationships create dependencies between residues. For example, we find on the test data described below that the correlation coefficient between spatial neighbors that are not also sequence neighbors (distance ≤3.5 Å) is 0.45. This is only slightly lower than the correlation coefficient between residues that are sequence neighbors (0.49). As there are more than three times as many spatial pairs of neighbors than sequence neighbors at this threshold it is reasonable from a modeling standpoint to use a model that respects *all* dependencies induced by spatial proximity, not only the dependencies induced by proximity along the backbone.

There are many papers using spatial neighborhood information of residues to predict-protein interaction sites (see e.g. [2, 13, 21, 28]). However, the spatial information of proteins was only integrated into the feature functions, but *not* represented in the model. For probabilistic models, the difference between the two ways to integrate spacial information is that in previous models the label of the *i*-th residue *Y*_*i*_ is conditional independent from the labels of other residues given data **X** and – in the case of linear CRFs or HMMs – given the labels of *Y*_*i*−1_ and *Y*_*i*+1_. Even when neighborhood information is only used for spatial smoothing of the labels, the intuitive advantage over, say, an SVM classifier that uses spatial neighborhood *in the features* but classifies each residue *independently*, is that not-patch-like candidate labelings are explicitly punished. In contrast, such an independent classifier-approach may have a tendency to predict individual interface residues ‘sprinkled’ around the protein surface [28].

For this reason, a general CRF seems to be more suitable for the task. However, inference for general CRFs is intractable. In this paper, pairwise conditional random fields (pCRFs) are utilized. Specializing general CRFs, only node cliques and edge cliques are taken into consideration in pCRFs. A pCRF retains most spatial information of proteins, can be specified with the same number of parameter as a line CRF and approximate inference remains feasible with the generalization of the Viterbi algorithm introduced here. Taking pattern from piecewise training methods [35], we disentangled the labels of nodes and edges to train the model.

In order to take advantage of a residue-wise score-based predictor, we model the protein surface by means of a pCRF, where the observation is solely a sequence of surface residue scores between 0 and 1 output by the predictor. We then utilize a generalized Viterbi algorithm and piecewise training. The resulting tool tries to enhance the predictor chosen on the surface of the protein under study. It is the aim of this paper to demonstrate effectiveness of this approach provided that the interface residue scores and the non-interface residue scores are appropriately distributed.

## Methods

We address the problem of improving residue-wise score-based predictors for protein interface residues as a node labeling problem for undirected graphs using the model class of conditional random fields (CRFs). Lafferty *et al.* [30] were the first who applied CRFs to the problem of labeling sequence data. Li *et al.* [28] used line CRFs to address the interaction site prediction. They have the advantage that the Viterbi algorithm well-known from decoding HMMs can be used to efficiently infer the most likely labeling sequence. Very useful and illustrative presentations on CRFs are given in [31, 32, 36, 37]. Above CRF-based models make the assumption that the label of one residue is conditionally independent of the labels of all other residues given the labels of the two adjacent residues in the protein sequence. To the best of our knowledge, we are the first to employ a graphical model that takes the spatial neighborhood of residues located on the protein surface into account.

This section is subdivided into three parts. We first explain how we model protein surfaces by pairwise CRFs. Then we introduce our new inference method. Finally, we elucidate our training method.

### Using conditional random fields to model protein surfaces

For every protein under study that has *n* surface residues, a pair of random vectors (**X**, **Y**) is considered. The vector **X** is the *observation* that represents the knowledge about this protein that is utilized in the prediction, e.g. the 3D structure of the target protein and a multiple sequence alignment together with homologs.

The vector **Y** is a random sequence of length *n* over the alphabet {I,N} that labels the index set {1,2,…,*n*}, which in turn is called the set of *positions* (of the surface residues). The label I represents interface residues, whereas the label N represents non-interface residues. {I,N}^*n*^ is the set of all label sequences of length *n* over {I,N}. We will also call them *assignments* as the term ‘label sequence’ may lead to confusion when applied below to subsets of {1,2,…,*n*} that are not contiguous sequences.

Let  be the *neighborhood graph*, where  is the set of positions,  is the set of edges that typically results from an atom-distance-based neighborhood definition for positions. We assume for convenience in notation that  has no isolated nodes. Cases with isolated nodes could trivially be reduced to cases without isolated nodes. Let  be the set of ’s *cliques*, which we refer to as *node cliques*. For a node clique  and an assignment **y** we denote by **y**_*c*_ the restriction of **y** to the positions belonging to the node clique *c*. For *c*={*i*} and *c*={*i*,*j*} we write *y*_*i*_ and  rather than **y**_{*i*}_ and **y**_{*i*,*j*}_.

The preceding notation is also used in the slightly more general case of partial label assignments to arbitrarily chosen subsets  of the set of positions . Formally, let  denote . Given two partial assignments  and  are identical on , the union  is well-defined.

The conditional distribution function of our pCRF (**X**, **Y**) with respect to the neighborhood graph  is defined as follows:
1

where **x** and **y** are arbitrarily chosen instances of the random observation **X** and the random label sequence **Y**, respectively, *Φ*_*c*_(*y*_*c*_,**x**)∈∙ () is the feature of the CRF located at the node clique *c* (again *Φ*_*i*_ and *Φ*_*i*,*j*_ simplify notation for *Φ*_{*i*}_ and *Φ*_{*i*,*j*}_), and *Z*(**x**) is the observation-specific *normalization factor* defined by
2

Let us call  the *score* of the label sequence **y** given the observation **x**.

A CRF is called a *pairwise CRF* (pCRF) if *Φ*_*c*_≡0, for all node cliques *c* larger than two. The remaining features *Φ*_*i*_ and *Φ*_*i*,*j*_ are referred to as *node features* and *edge features*, respectively. Thus, every position  and every edge  is represented by the pair (*Φ*_*i*_(N,**x**),*Φ*_*i*_(I,**x**)) and by the quadruplet (*Φ*_{*i*,*j*}_ (N,N,**x**),*Φ*_{*i*,*j*}_(I,N,**x**),*Φ*_{*i*,*j*}_(N,I,**x**),*Φ*_{*i*,*j*}_(I,I,**x**)).

Following [30], we assume moreover that each node feature and each edge feature is a sum of weighted base features. More precisely, for every position  and every edge  we assume representations


where **y**∈{I,N}^*n*^ and **x** is an observation. The two real vectors
3

need to be calculated in a training phase.

In the most general sense, protein characteristics are real-valued evaluations of positions and pairs of adjacent positions (edges of the neighborhood graph), respectively, that are correlated with our position labeling problem. We use a standard step function technique to obtain base features from protein characteristics, rather than taking the raw values of the characteristics. To make our paper self-contained, let us describe this technique for short.

A protein characteristic depends on the observation and either a node or an edge. Each protein characteristic, such as e.g. the relative solvent-accessible surface area of a residue, is transformed into several binary features by binning, i.e. we distinguish only a few different cases rather than the whole range of the characteristic. Assuming the common case of real-valued characteristics, the bins are a partition of the reals into intervals. The use of this discretization allows to approximate any shape of dependency of the labels on the characteristics, rather than assuming a fixed shape such as linear or logarithmic.

*From protein characteristics for positions to node features.* We subdivide the range of the characteristics *C* into say *γ* intervals, where *γ* is at least two. Let *s*_1_<*s*_2_<…<*s*_*γ*−1_ be the corresponding interval boundaries. It is reasonable to take *s*_*ι*_ as the *ι*/*γ*-quantile of the empirical distribution of *C* for non-interface residues, where *C*(*i*,**x**)∈(*s*_0_,*s*_*γ*_]. Then we define for each position  the following 2*γ* base features associated with the position characteristics *C*.
4

where *y*=N,I, and *ι*=0,1,…,*γ*−1 and *s*_0_:=−*∞*,*s*_*γ*_:=*∞*.

*From protein characteristics for edges to edge features.* Let *D* be the characteristics. Analogous to the previous case, we then obtain for each edge  the following 4*γ* base features associated with *D*, where *y*,*y*^′^∈{N,I} and *ι*=0,1,…,*γ*−1.
5

In both cases we set *γ*=5.

### Devising a generalized Viterbi algorithm for pCRFs

The problem of finding a most probable label sequence **y**^∗^ given an observation **x** is **NP**-hard for general pCRFs [31]. In this subsection we present a heuristic that approximately solves this problem.

To this end, we first devise an algorithm, which we call *generalized Viterbi algorithm*. It computes an optimal label sequence, where the posterior probability of **y**^∗^ given **x** is maximized. Unfortunately, its run-time is in too many cases not acceptable. That is why we transform it in a second step into a feasible, time-bounded approximation algorithm.

#### The generalized Viterbi algorithm

Let  be the neighborhood graph underlying the protein under study. For any assignment (label sequence) **y** and any subset  of , let  denote the partial assignment of **y** with respect to . (This is in line with the notation **y**_*c*_ (*c* a position clique) introduced earlier in this study).

If  are pairwise disjoint position sets, the assignment for  canonically resulting from assignments  is denoted by . For , the score  is defined by


Then the problem of determining a most probable label sequence **y**^∗^ given an observation **x** can be reformulated as


This is the case, because it suffices to consider the score.

To put this into practice, we devised an algorithm we call generalized Viterbi. On the one hand, it is analogous to the classical Viterbi algorithm. On the other hand, there is a major difference. In our case there is no canonical order in which the positions of  are traversed. Having explained our algorithm for any order, we show how to calculate a fairly effective one. In what follows, we assume that the positions not yet touched are held in a dynamic queue. Those positions having already left the queue form the *history set*.

**Figure 1:**
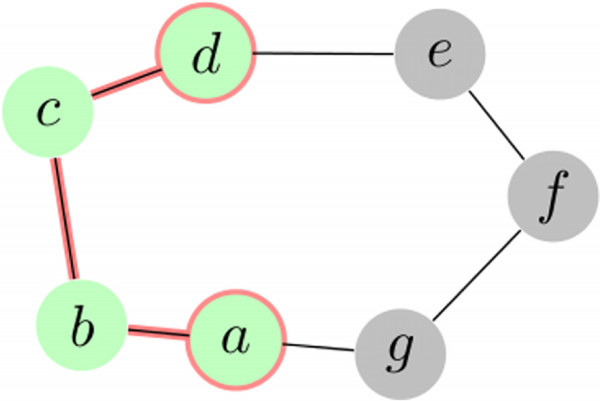
**Example history set**  **having boundary**  **.**

Assume that the subgraph of  induced by  has connected components , …, . For *μ*=1,2,…,*m*, let  be the so-called *boundary component* associated with  defined by . The complement  is the *interior* of the *μ*-th history component. See Figure 1 for an example.

For assignments  of the boundary components , the Viterbi variables  are defined as
6

The Viterbi variables can be represented as a set of tables, one table of size  for each boundary component . In the case where a boundary component is empty the table reduces to a single number.

At any stage, the algorithm stores the connected components , …,  of the current history set , the corresponding boundary components , …, , and Viterbi variable values  where  range over all possible assignments of corresponding boundary component. We store for every assignment on the boundary, a maximizing interior assignment. This assignment is the argmax of (6) but is determined with the dynamic programming recursions defined below. Let us call these data the current state of the algorithm. It mainly consists of record sets indexed by the boundary labelings.

At the very beginning the queue contains all positions, the history set  and the corresponding boundary component  are empty. As long as the position queue is not empty, the top element *v* is extracted and the state is updated as follows.

**Figure 2:**
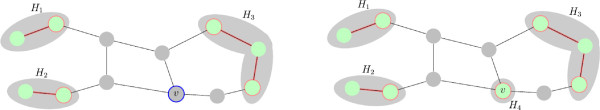
**Computing the connected components of the new history set - case one.**

**Figure 3:**
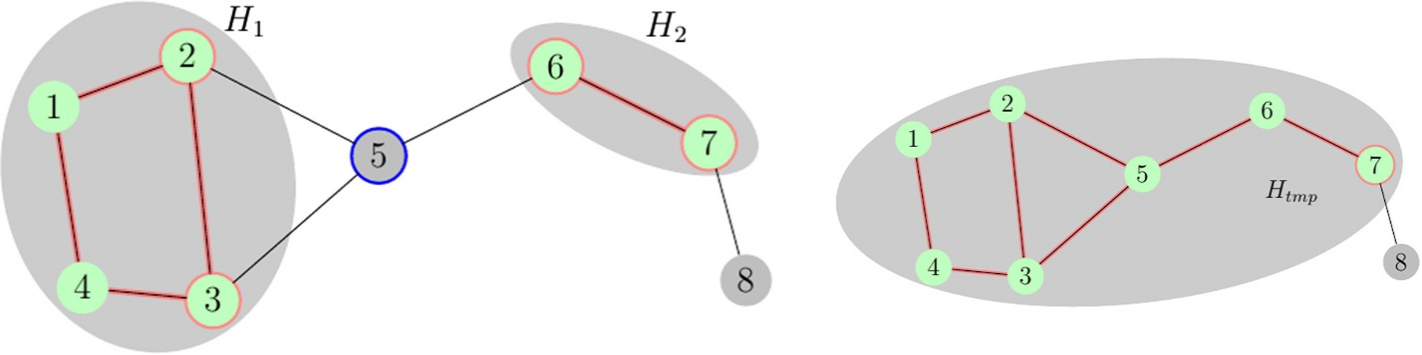
**Computing the connected components of the new history set - case two.**

Adjoining *v* to the history set , there are two cases to distinguish. Either position *v* is not adjacent to any other position of any old boundary component (see Figure 2) or adjoining position *v* to  results in adding it to some connected component of the old history set or even merging together two or more of them (see Figure 3).

In the first case we simply have to take over all the old connected components, boundary sets and Viterbi variables. Moreover, we perform the instructions


In the second case position *v* is adjacent to some boundary components, say . Then the old history components  and the current position *v* are merged together:


The other history set components and corresponding Viterbi variables are not affected.

For *μ*=*m*^′^,*m*^′^+1,…,*m*, let  be the set of all positions out of  that are no longer boundary nodes after having adjoined *v* to the history set. The nodes in  are removed from the boundary  after the iteration. Let  be the complement of  in . By inspecting the edges incident to the current position *v*, all these sets can be computed in linear time.

The new boundary set  is then either  or , where it can be checked in linear time whether or not *v* is a new boundary position.

We are now in a position to calculate the new Viterbi variables , where  ranges over all assignments of the new boundary set .

If  then


**Figure 4:**
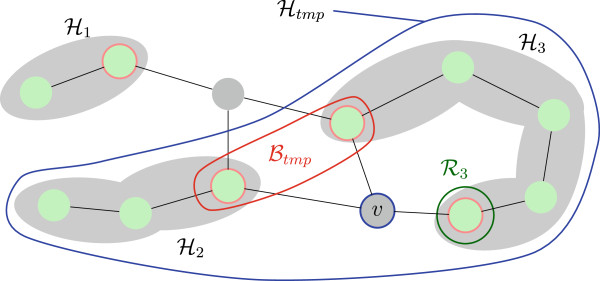
**Viterbi recursion step in case**  **.** After adding node *v* to the history set,  and  will be replaced by . In this example, for every assignment of the new boundary  the score of  is maximized by varying over the assignments of *v* and  and using the Viterbi variables of  and .

Here, any assignment of a node set is assumed to implicitly define assignments for any subset thereof. Figure 4 illustrates this case of the recursion step. If, however, , then

Finally, the interior labeling is stored, where the maximum is attained. The algorithm terminates after the last node *v* from  has been processed. In the typical case, where the graph is connected, at termination .

The running time of the algorithm is , where *b* is the size of the largest boundary set and *n* is the number of surface residues. We call this algorithm *generalized Viterbi* algorithm as for the case of a graph that is a linear chain 1−2−3−⋯−*n* of nodes using the node order 1,2,…,*n* the Viterbi variables we define are the same as in the standard Viterbi algorithm for HMMs. In the case of a graph that is a tree, this algorithm specializes to the Fitch algorithm or an argmax-version of Felsenstein’s pruning algorithm when a leaf-to-root node order is chosen after rooting the tree at an arbitrary node. In both special cases the boundary sets always have size at most 1. The tree example also motivate the use of several history sets at the same time: using a single history set only, one would not be able to achieve a linear running time on trees.

#### A heuristic based on the generalized Viterbi algorithm

First, it is vital for our generalized Viterbi algorithm to keep the size of the boundary sets small. A good position order is here of great importance. The algorithm starts by choosing a vertex of minimal degree. When determining the next position to be dequeued, the algorithm selects a boundary node such that the number of incident edges leading to nodes not belonging to any current history set is minimal. In an arbitrarily chosen order these nodes are dequeued next.

Second, the space demand is reduced by restricting the number of boundary labelings admitted. Starting from the available labelings of the current history set, the percentage of the reachable boundary labelings of the successor history that will be discarded is calculated. Then the corresponding percentile is estimated. To this end, a sufficiently large sample of possible labelings of the new boundary set is drawn, the Viterbi variables are computed, and the corresponding sample percentile is taken. Finally, only those boundary labelings of the new history set are retained whose Viterbi variables exceed this percentile.

That way we compute near-optimal solutions good enough for our purposes within feasible computation time.

### Piecewise training for pCRFs

Let


be the independent identically distributed training sample. For every *μ*=1,2,…,*m*, let  and  be the set of positions and edges in the neighborhood graph associated with **x**_*μ*_, let  be the number of positions of the *μ*-th training example and let  be the set of all possible label sequences of this graph.

This data set is unbalanced as there are many more non-interface positions as interface positions. As customary for other machine learning approaches such as support vector machines and artificial neural networks [28], we here manipulated the ratio of positive and negative example positions for training in order to obtain reasonable results.

We have amplified the influence of the positive examples, rather than selecting various sets of training data by deleting negative ones as done in [28].

Let *ν*_*I*_, *ν*_*N*_, *ν*_*II*_ and *ν*_*NN*_ be the number of interface positions, the number of non-interface positions, the number of interface-interface edges, and the number of non-interface-non-interface edges in , respectively. Then we define the following two *amplifier functions* for all positions *i* and for all edges {*i*,*j*} of the *m* neighborhood graphs resulting from the training data .


To uniformly govern the influence of the amplifiers, we introduce an *amplifier control parameterη*_3_∈ [ 0,1].

We set up our two log-likelihood objective function by


where ideally for each *μ*=1,2,…,*m*

is the training-instance-specific normalization factor.

Unfortunately, maximizing this objective function in general is algorithmically intractable. Taking pattern from Sutton *et al.* [35] who introduced what they called piecewise training, we deal with this problem by disentangling the labels of nodes and edges. For *μ*=1,2,…,*m*, a *non-coherent labeling* of the neighborhood graph  is any mapping that assigns to every position  and every edge  a label **y**_*v*_∈{I,N} and a pair of labels **y**_*e*_∈{I,N}^2^, respectively.

We then replace *Z*(**x**^(*μ*)^,*η*_3_) by


as normalization factor. This makes the optimization problem computationally feasible.

The L-BFGS method [38] is used to solve it. That way we obtain the coefficient vectors **α** and **β** (see Equations 3), which depend on the amplifier control parameter *η*_3_∈ [ 0,1].

To mitigate the negative consequences of disentanglement, we use a *correction factorδ*≥1. For any characteristics *D* and *ι*=0,1,…,*γ*−1, the weights of the bases edge features  and  (see Equation 5) are all multiplied by *δ*. Thus a change in classification along an edge is additionally penalized. The correction factor *δ* is set best between 1.15 and 1.25.

For our implementation of the training, we used the Java CRF package from Sunita Sarawagi at http://crf.sourceforge.net/.

## Results and discussion

In this section we demonstrate effectiveness of our pCRF-based protein surface model to enhance residue-wise score-based predictions of protein-protein interfaces. For the sake of ensuring reliability of the methods we used three data sets. The first one is *PlaneDimers* due to Zellner *et al.* [25], the second one is the list of 1276 two-chain-proteins published by Keskin *et al.* [39], which was used by Li*et al.* [28] to test their linear-chain CRF. Third, we used a non-redundant data set containing 22604 unique interface structures very recently compiled by Cukuroglu *et al.* and published in [40].

The data set *PlaneDimers* is less known than the data due to Keskin *et al.*. It consists of redundancy-free homodimers with flat protein-protein interfaces. Zellner *et al.* [25] developed an SVM, called *PresCont*, that assigns to each residue on the protein surface a score between 0 and 1, which we refer to as *PresCont* score in the sequel. The larger the score, the more likely the residue belongs to the interface. Zellner *et al.* made the prediction by thresholding the score. The *PresCont* server and the data list *PlaneDimers* are publicly available (see http://www-bioinf.uni-regensburg.de/).

In the first subsection we describe two sets of experiments performed with synthetic data, one on *PlaneDimers* [25], the other one on the list published by Keskin *et al.* [39]. In both cases we independently assign to each surface position a random score drawn according to two different parametrized sequences of *β*-distributions Beta(*α*_I_(*ς*)*β*_I_(*ς*)) and Beta(*α*_N_(*ς*)*β*_N_(*ς*)), one for the interface sites determined by the reference labeling, the other one for the non-interface positions. The parametrized values *α*_I_(*ς*), *α*_N_(*ς*), *β*_I_(*ς*) and *β*_N_(*ς*) determining the two sequences of distributions are chosen such that the following conditions are satisfied. The mean values *e*_I_>*e*_N_ are the average *PresCont* scores on interface sites and non-interface sites of all chains from *PlaneDimers*. The variances  and  are equal to  and , where  and  are the corresponding variances of the *PresCont* score, and *ς*∈{0.8,0.9,1.0,1.1,1.2} models the precision of the synthetic score. The deciding feature of all these distributions is that they are *unimodal*. The result of the subsection is that enhancement works for unimodal score distributions.

The second subsection is about a synthetic data experiment on a new data set due to Cukuroglu [40]. Here we follow the line of the first subsection except for the fact that we restrict ourselves to signal precision *ς*=1.0.

**Figure 5:**
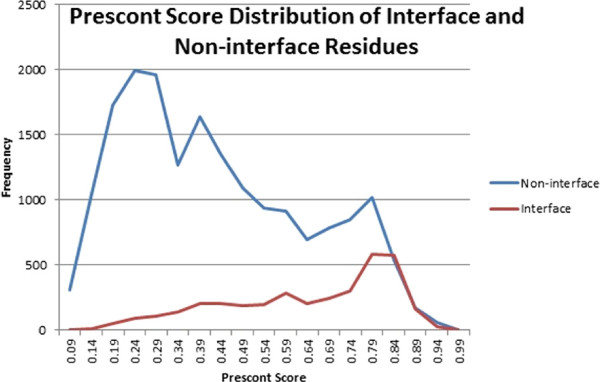
**The distribution of the** ***PresCont*** **score for complexes from the data set** ***PlaneDimers*** **.**

In the third subsection we study the *PresCont* scores for two-chain protein complexes from the data set *PlaneDimers*. According to Figure 5, the *PresCont* score for non-interface residues is far from being unimodal. However, if one restrict oneself to the part above a threshold in the neighborhood of 0.5 and larger, one may ask whether enhancement restricted to that domain will works. The subsection answers this question in the affirmative. Having chosen a threshold as described above, one can improve the classification with respect to this threshold as follows. Take over the prediction for scores below the threshold and reclassify the residues the scores of which are above by means of the pCRF-based enhancer.

In general, observations **x** could encompass a PDB file, which in particular determines the 3D-structure of the protein, together with an MSA that models evolutionary aspects. In our case an observation solely consists of the *PresCont* score sequence or of the sequence of synthetic scores for the surface residues. Formally, every observation **x** is equal to a vector (*ζ*_1_,*ζ*_2_,…,*ζ*_*n*_)∈ [ 0,1]^*n*^.

There are several neighborhood notions for residues, surface/core definitions and interface determinations in the literature. When studying the data set *PlaneDimers*, we follow [25]. In the case of the list due to Keskin *et al.* [39], the definitions according to [28] are used. Finally, when studying complexes taken from the data set published in [40], we take the following definitions. The RASA value of a surface residue is at least 15*%* (see [28]). Two residues are defined as contacting if the distance between any two of their atoms is less than the sum of the corresponding van der Waals radii plus 0.5 Å (see [40]).

Anyway, according to Keskin *et al.* [39] we define the *distance* of two residues on one and the same chain as the distance of their major carbon atoms. We then say that one residue is *nearby* another residue, if they are at distance below 6 Å. (Note that usually residues adjacent on backbone are at distance of less than or equal to 3.5 Å). This definition in turn is the basis of the neighborhood graph  underlying the pCRF. Two surface positions are joined together by an undirected edge if and only if the corresponding residues are nearby ones.

Our pCRF-based enhancer utilizes one position characteristic and two edge characteristics on the basis of the standard step function method explained in the Methods section. If **x**=(*ζ*_1_,*ζ*_2_,…,*ζ*_*n*_)∈ [ 0,1]^*n*^ is the observation associated with the protein under study, and if  is the neighborhood graph, then for every position  and every edge  we set


To enhance predictions obtained by thresholding, solely information coming from the residue neighborhood relations on the surface is additionally used.

In order to be able to calculate the performance measure of *area under the ROC curve* (AUC) for our pCRF-based enhancer on synthetic scores, we proceed as follows. For each edge , we replace the local feature value *Φ*_*i*,*j*_(I,I,**x**) by *κΦ*_*i*,*j*_(I,I,**x**), where *κ*∈(0,*∞*).

We enhance residue-wise score-based predictors only on the protein surface. In our synthetic data experiments there is no predictor available for core residues. For proteins taken from the data list published by Keskin *et al.* [39] it happens that interface sites belong to the core. That is why we use what we call *Surface AUC RatioΓ* of the enhancer as our performance measure for our synthetic data experiments.


If *Γ* is greater than 1, the enhancement was successful. The larger *Γ*, the greater success.

To estimate performance measures, we applied 5-fold cross-validation experiments.

A fully built-out pCRF-based tool box for modeling protein surfaces needs to comprise all the standard algorithms as e.g. forward-backward techniques, marginalization and posterior decoding known for HMMs and linear-chain CRFs. To begin with, in the fourth subsection we explain how to put a variant form of the forward algorithm and posterior decoding for pCRFs into practice.

### Simulating unimodal scores of various precisions

We estimated means *e*_I_ and *e*_N_ and variances  and  of the *PresCont* score on interface sites and noninterface positions of *PlaneDimers*, respectively, as follows.
7

We randomly chose 120 instances under the uniform distribution from the data set published by Keskin *et al.* [39] to perform our experiments. Let us refer to this set as *KL-subset* in the sequel. (It is accessible at http://ppicrf.informatik.uni-goettingen.de/index.html).

Zellner *et al.* [25] used the following determinations. A residue is defined to be part of the protein surface, if its relative solvent-accessible surface area is at least 5*%* [17]. A surface residue is said to constitute an *inter-facial contact*, if there exists at least one atom of this residue which has a van-der-Waals-sphere at a distance of at most 0.5 Å from the van-der-Waals sphere to any atom from a partner chain residue [39].

Based on [3, 12, 15, 20, 41], Li *et al.* [28] assume an inter-facial contact of a residue on a chain is assumed to be there, if any heavy atom of this residue is at distance of at most 5 Å from any heavy atom from a partner chain. The relative solvent-accessible surface area of surface residue is at least 15*%*.

We independently assigned to each interface surface residue of the two data sets a random score between zero and one according to the *β*-distribution Beta(*α*_I_(*ς*)*β*_I_(*ς*)), and to every non-interface surface residue a score according to Beta(*α*_N_(*ς*)*β*_N_(*ς*)), where the score precision *ς* satisfies
8

and the parameters *α*_I_(*ς*),*β*_I_(*ς*),*α*_N_(*ς*),*β*_N_(*ς*) were chosen such that
910

The Surface AUC Ratios of the enhancer compared with the threshold predictor on *PlaneDimers* and the *KL*-subset are displayed in Table 1. There is an improvement of 8.4*%*−9.3*%* on *PlaneDimers* and of 3.2*%*−5.0*%* on the KL-subset.

**Table 1:** **Classification results on** ***PlaneDimers*** **and the KL-subset, where the** ***β*** **-distributions according to which the synthetic scores were drawn are defined by Equations** 7**,** 8**,** 9 **and** 10

***PlaneDimers***	Score precision***ς***	0 ***.***8	0 ***.***9	1 ***.***0	1 ***.***1	1.2
	Surface AUC ratio *Γ*	1.084	1.091	1.093	1.089	1.093
*Kl-subset*	Signal precision *ς*	0.8	0.9	1.0	1.1	1.2
	Surface AUC ratio *Γ*	1.032	1.039	1.045	1.045	1.050

Depending on the variances determined by *ς*, the enhancer increases the AUC referred to the protein surface by 8.4%-9.3%. on *PlaneDimers*, and by 3.2%-5.0% on the KL-subset.

Moreover, we compared individual classification results obtained by thresholding the scores with pCRF-based enhanced predictions. Because of the fact that the specificity of the threshold predictor can be easily changed by manipulating the threshold, we proceeded as follows. For every score precision, the pCRF-based enhancer has a well-defined specificity referred to the surface residues. We then chose the threshold such that the specificity of the threshold predictor is close to that of the enhancer. The results are shown in Table 2. The sensitivity is increased by 53*%*−67*%* on the data set *PlaneDimers* and by 14*%*−22*%* on the KL-subset.

**Table 2:** **Comparing the enhancer with the threshold classifier of approximately equal specificity on synthetic scores assigned to surface residues of protein complexes taken from the data set** ***PlaneDimers*** **and the KL-subset**

Data Set	Score Precision***ς***	Classifier	Specificity	Sensitivity	MCC
	0.8	Threshold Predictor	0.9672	0.2562	0.3253
		Enhancer	0.9666	0.4281	0.4911
*PlaneDimer*	0.9	Threshold Predictor	0.9618	0.2556	0.3077
		Enhancer	0.9624	0.4086	0.4610
	1.0	Threshold Predictor	0.9611	0.2428	0.2912
		Enhancer	0.9612	0.3872	0.4379
	1.1	Threshold Predictor	0.9681	0.2100	0.2753
		Enhancer	0.9677	0.3307	0.4045
	1.2	Threshold Predictor	0.9649	0.2100	0.2648
		Enhancer	0.9647	0.3213	0.3854
	0.8	Threshold Predictor	0.9568	0.2936	0.3549
		Enhancer	0.9577	0.3586	0.4210
*KL-subset*	0.9	Threshold Predictor	0.9533	0.2843	0.3369
		Enhancer	0.9531	0.3290	0.3820
	1.0	Threshold Predictor	0.9570	0.2559	0.3152
		Enhancer	0.9571	0.2971	0.3591
	1.1	Threshold Predictor	0.9615	0.2279	0.2949
		Enhancer	0.9614	0.2743	0.3459
	1.2	Threshold Predictor	0.9604	0.2199	0.2828
		Enhancer	0.9599	0.2516	0.3175

Table 1 and Table 2 justify the following conclusion. Enhancing the threshold prediction by our pCRF works provided that the distributions of the interface scores as well as the non-interface scores are unimodal. The enhancement for the data set *PlaneDimers* is larger than for the KL-subset. This might be caused by the plain interface geometry of the complexes taken from *PlaneDimers*.

### Utilizing the new data set due to Cukuroglu [40]

As in the case of the KL-subset, we randomly chose 60 dimers. We refer to the resulting list as *CGNK-subset*. Having assigned synthetic scores according to Equations 7, 9 and 10, where *ς*=1.0, we compared individual classification results obtained by thresholding the scores with pCRF-based enhanced predictions in exactly the same way as we did for the KL-subset. The results are shown in Table 3. The sensitivity is increased by 22*%*.

**Table 3:** **Comparing the enhancer with the threshold classifier of approximately equal specificity on synthetic scores assigned to surface residues of protein complexes taken from the CGNK-subset**

Classifier	Specificity	Sensitivity	MCC
Threshold predictor	0.9399	0.3782	0.3387
Enhancer	0.9400	0.3104	0.2767

A main finding of Cukuroglu [40] relevant to protein-protein interface prediction is, that the average interface RASA value is greater than 40*%*. Since our method is designed to improve performance of a given residue-wise predictor, using this result is not in the scope of this paper. However, a CRF-based predictor integrating features for cliques of size greater than 2 is not beyond the range of current algorithmic capabilities. In such a model a feature set that discretizes the mean RASA value of cliques is promising.

### Enhancing the *PresCont*server prediction on *PlaneDimers*

For the sake of completeness, we shortly review the residue characteristics used by *PresCont*.

#### Relative solvent-accessible surface area

For any residue *a*, the *solvent-accessible surface area***asa**(*a*) can be computed by e.g. the software library BALL [42]. Most of the classifiers known from the literature utilize this characteristic (see [43]). For *PresCont* the *relative solvent-accessible surface area* according to
11

is taken into operation, where **asa**_max_(*a*) is the maximally possible accessible surface area of residue *a* [44].

#### Hydrophobicity

Many interfaces possess a hydrophobic core surrounded by a ring of polar residues [45, 46]. In order to reduce noise, in [25] the contribution of hydrophobic patches rather then the influence of individual residues is utilized.

#### Residue conservation

Measures of this type utilized in [25] are the Shannon entropy and the relative Shannon entropy of empirical residue distributions in MSA columns. As an alternative, empirical expectations of BLOSUM-based similarities are taken for them.

#### Scores of local neighborhoods

They are evaluated by means of log-odd ratios of neighboring residue pair frequencies in interfaces as opposed to residue pair frequencies on complementary protein surface areas. The resulting scores are averaged both over the neighborhood of the positions under study and the rows of the MSA associated with the protein.

On the basis of Figure 5 we enhanced *PresCont* for thresholds *θ*∈ [ 0.500,0.625]. The decisive factor for this choice is that the *PresCont* score distributions for interface sites as well as non-interface positions above *θ* are “sufficiently close to” unimodal distributions. For every such *θ*, we set all scores less than or equal to *θ* to zero and then left the classification of all surface residues to the pCRF modified as follows. The residues of score zero are not taken into account when it comes to discretizing the protein characteristics (see Equations 4 and 5). Let us call this *enhancing aboveθ*.

**Figure 6:**
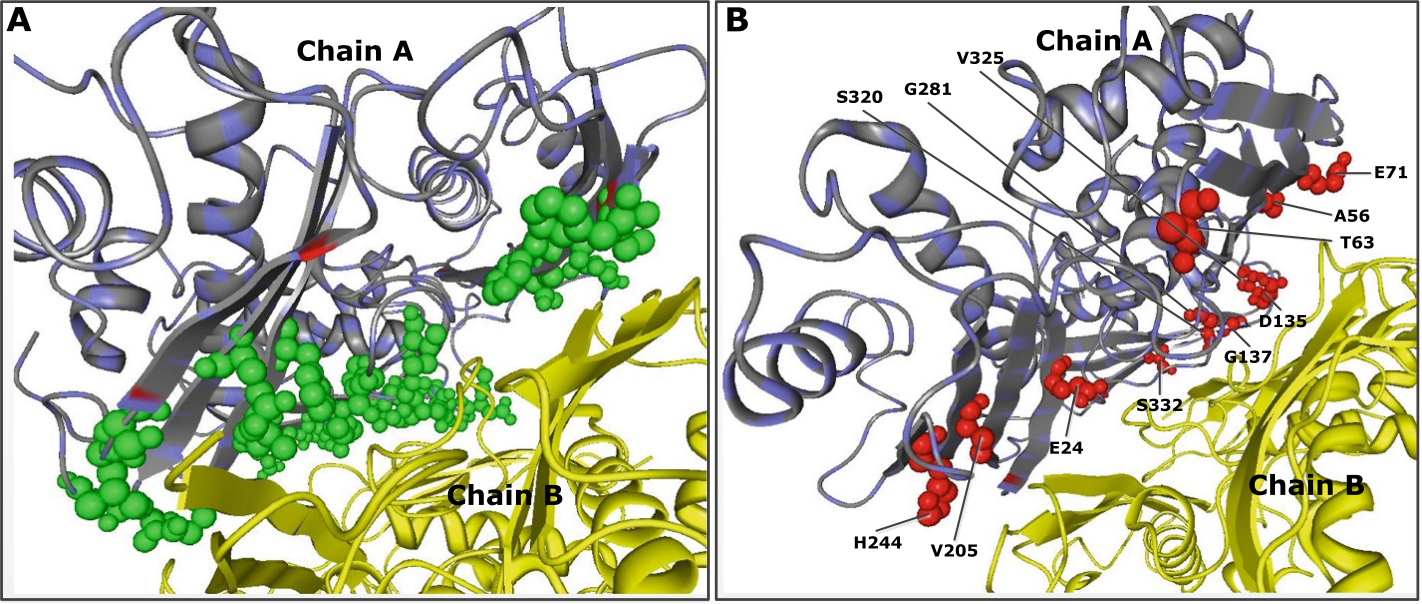
**Comparison of enhancer and** ***PresCont*** **service of same specificity on the protein with PDB-Entry 1QM4.** **(A)** Green spheres on the left show the interface surface residues correctly predicted by both tools. **(B)** Red spheres on the right indicate *additional* true positives of the enhancer.

To evaluate improvements we proceeded as when compiling Table 2. For every threshold *θ* under consideration another threshold *θ*^′^ was chosen such that thresholding at *θ*^′^ has the same specificity as enhancing above *θ*. The results are displayed in Table 4 and visualized for an individual protein by Figure 6. According to Table 4 the increase in sensitivity ranges from 4*%* to 7*%*. The true-positive predictions on the surface of the protein with PDB-Entry 1QM4 are compared in Figure 6, where again the specificity of the two classifiers is the same.

**Table 4:** **Enhancing above various thresholds on** ***PlaneDimers*** **, where** ***PresCont*** **’s threshold was chosen such that the specificity approximately equals that of enhancing**

	tp	tn	fp	fn	Spec.	Sen.	MCC	
Enhancing above 0.500	2181	23182	4145	1414	0.848	0.607	0.362	
*PresCont*	2100	23197	4130	1495	0.849	0.584	0.346	
Enhancing above 0.525	2303	22917	4410	1292	0.839	0.641	0.373	
*PresCont*	2206	22912	4415	1389	0.838	0.614	0.353	
Enhancing above 0.550	2507	22103	5224	1088	0.809	0.697	0.375	
*PresCont*	2419	22102	5225	1176	0.809	0.673	0.358	
Enhancing above 0.575	2560	21992	5335	1035	0.805	0.712	0.380	
*PresCont*	2463	21915	5412	1132	0.802	0.685	0.358	
Enhancing above 0.600	2379	22685	4642	1216	0.830	0.662	0.376	
*PresCont*	2253	22780	4547	1342	0.834	0.627	0.356	
Enhancing above 0.625	2287	23044	4283	1308	0.843	0.636	0.376	
*PresCont*	2136	23049	4278	1459	0.843	0.594	0.346	

The sensitivity increased that way by 4%-7%. For every pair of experiments, the number of true negatives (tn), false negatives (fn), false positives (fp) and true positives (tp) are displayed.

### Discussing posterior decoding

As in the case of linear-chain CRFs, the generalized Viterbi algorithm can be transformed into a variant form of the forward algorithm. It might be the case that the following additional problem arises.

Let *v*_1_,*v*_2_,…,*v*_*n*_ be the ordering in which the positions of  are traversed by the algorithm, and let  denote the set of position indices *i*<*n* such that *v*_*i*_ is not an element of the boundary  of the history set  at stage *i*. If  is not empty, we encounter an obstacle when it comes to sampling label sequences. For , position *v*_*i*_ is not labeled in the course of the sampling procedure. That is why we augment the neighborhood graph  so that those positions no longer exist, all predictions remain unchanged, and the order of magnitude of the running time is not increased. To this end, we complement the ordering *v*_1_,*v*_2_,…,*v*_*n*_ as follows. For every , we insert a new node  between *v*_*i*_ and *v*_*i*+1_. Having extended the neighborhood graph by these nodes not being associated with residue positions of the protein under study and by new edges , where for  and  the above mentioned obstacle is eliminated without any influence on the prediction and the order of magnitude of the running time.

Proceeding now in a way analogous to the classical case, in every formula that is a building block of the generalized Viterbi algorithm the following two steps of replacement need to be performed.

First, for every position , every edge , every label *y*_0_∈{I,N}, and every label pair (*y*_1_,*y*_2_)∈{I,N}^2^, we replace *Φ*_*i*_(*y*_0_,*x*) with exp(*Φ*_*i*_(*y*_0_,*x*)), and *Φ*_{*i*,*j*}_(*y*_1_,*y*_2_,*x*) with exp(*Φ*_{*i*,*j*}_(*y*_1_,*y*_2_,*x*)).

Second, we replace sums with products and then maxima with sums.

Thus we obtain as analogues of the Viterbi variables  defined by Equation 6 what we call component forward variables .

If  and  are the connected components of the history set  and the corresponding boundary set  at stage *i*∈{1,2,…,*n*}, respectively, then the forward variable at stage *i* with respect to a boundary assignment  is defined as


For any assignment  (*i*>1), the forward variable  is a nontrivial linear combination of forward variables , where  ranges over some assignments of the boundary set  at stage *i*−1. Analogous to the linear-chain case, a random backward walk through a state graph, with all possible assignments  (*i*=*n*,*n*−1,…,1) being the set of nodes, results in a random labeling of the positions, where each labeling is drawn with its posterior probability.

This sampling technique allows the efficient calculation of posterior probabilities at nodes and edges in a straightforward manner.

## Conclusions

Residue-wise score-based threshold predictors of protein-protein interaction sites assign to each residue of the protein under study a score. The classification is then made by thresholding the score. In case of using probabilistic data models, the parameters of the threshold predictor have been learned on a training data set in advance.

We have demonstrated that such threshold predictors can be improved by pCRF-based enhancers given the shape of the interface surface score distribution and the non-interface surface score distribution with respect to the training set resemble the shape of unimodal distributions. Besides the surface residue scores, only the spatial neighborhood structure between the surface residues of the protein under study is taken into account. Thus, the improvement can be attributed to our model. In addition to the precision of the scores, the amount of improvement depends on the 3D-complexity of the interfaces to be predicted. To this end, three sets of experiments with synthetic surface residue scores for protein complexes randomly chosen from the data set *PlaneDimers* compiled by Zellner *et al.* [25] and from the lists published by Keskin *et al.* [39] and Cukuroglu *et al.* [40].

The enhancement is structurally based on the following model property of pCRFs in contrast to residue-wise predictors. Though the scores of near-by residues may be correlated, labeling a position as interface or non-interface by thresholding the score does *not* influence the classification of its neighbors. When using pCRFs, this is the case.

The pCRF-based enhancer is also applicable, if the score distributions are only unimodal over a certain sub-domain. The improvement is then restricted to that domain. Thus we were able to improve the prediction of the *PresCont* server devised by Zellner *et al.* on *PlaneDimers* [25].

The prediction is made on grounds of a generalized Viterbi inference heuristic. As for training, we developed a piecewise training procedure for pCRFs, where the enhancer needs to be trained on data originating from the same source as the training data of the threshold predictor to be improved.

A prototypical implementation of our pCRF-based method is accessible at http://ppicrf.informatik.uni-goettingen.de/index.html.
